# Supplementary material for: Multi‐dataset identification of innovative feature genes and molecular mechanisms in keratoconus
Source: J Cell Mol Med. 2024 Sep 19;28(18):e70079. doi: 10.1111/jcmm.70079 (PMC11412914; doi:10.1111/jcmm.70079)
Supplement: Supplementary file 2 — Material S2. [file JCMM-28-e70079-s002.zip › 7.GSEA analysis/1.ARL11/KEGG_GLYCINE_SERINE_AND_THREONINE_METABOLISM.html]

Details for gene set KEGG\_GLYCINE\_SERINE\_AND\_THREONINE\_METABOLISM[GSEA]

|  || Dataset | ARL11.group.cls#h\_versus\_l |
| Phenotype | group.cls#h\_versus\_l |
| Upregulated in class | h |
| GeneSet | KEGG\_GLYCINE\_SERINE\_AND\_THREONINE\_METABOLISM |
| Enrichment Score (ES) | 0.3501037 |
| Normalized Enrichment Score (NES) | 1.1757447 |
| Nominal p-value | 0.1904762 |
| FDR q-value | 1.0 |
| FWER p-Value | 0.993 |
Table: GSEA Results Summary

  

Fig 1: Enrichment plot: KEGG\_GLYCINE\_SERINE\_AND\_THREONINE\_METABOLISM      
 Profile of the Running ES Score & Positions of GeneSet Members on the Rank Ordered List

  

| SYMBOL | TITLE | RANK IN GENE LIST | RANK METRIC SCORE | RUNNING ES | CORE ENRICHMENT || 1 | DMGDH | na | 926 | 0.398 | 0.0614 | Yes |
| 2 | MAOB | na | 1064 | 0.382 | 0.1421 | Yes |
| 3 | AOC2 | na | 1449 | 0.342 | 0.2067 | Yes |
| 4 | BHMT | na | 1693 | 0.320 | 0.2705 | Yes |
| 5 | ALAS2 | na | 2163 | 0.285 | 0.3200 | Yes |
| 6 | GLYCTK | na | 3983 | 0.201 | 0.3121 | Yes |
| 7 | AGXT2 | na | 4162 | 0.195 | 0.3501 | Yes |
| 8 | GLDC | na | 5741 | 0.144 | 0.3365 | No |
| 9 | PSPH | na | 8507 | 0.083 | 0.2751 | No |
| 10 | DLD | na | 9166 | 0.072 | 0.2721 | No |
| 11 | GATM | na | 9750 | 0.064 | 0.2694 | No |
| 12 | GAMT | na | 11997 | 0.036 | 0.2127 | No |
| 13 | MAOA | na | 12281 | 0.034 | 0.2119 | No |
| 14 | SHMT1 | na | 14696 | 0.014 | 0.1454 | No |
| 15 | SHMT2 | na | 14742 | 0.013 | 0.1470 | No |
| 16 | SRR | na | 16244 | 0.002 | 0.1042 | No |
| 17 | PSAT1 | na | 17729 | -0.008 | 0.0632 | No |
| 18 | CHDH | na | 19152 | -0.018 | 0.0262 | No |
| 19 | SDS | na | 22931 | -0.049 | -0.0717 | No |
| 20 | PHGDH | na | 23647 | -0.058 | -0.0796 | No |
| 21 | AGXT | na | 24023 | -0.063 | -0.0765 | No |
| 22 | CTH | na | 24353 | -0.067 | -0.0712 | No |
| 23 | PIPOX | na | 25663 | -0.085 | -0.0901 | No |
| 24 | GCAT | na | 26073 | -0.091 | -0.0817 | No |
| 25 | ALAS1 | na | 26487 | -0.098 | -0.0718 | No |
| 26 | CBS | na | 28775 | -0.151 | -0.1043 | No |
| 27 | AMT | na | 29406 | -0.169 | -0.0850 | No |
| 28 | GNMT | na | 29855 | -0.183 | -0.0574 | No |
| 29 | SARDH | na | 31504 | -0.239 | -0.0519 | No |
| 30 | AOC3 | na | 32726 | -0.302 | -0.0202 | No |
| 31 | DAO | na | 33410 | -0.352 | 0.0380 | No |
Table: GSEA details [plain text format]

  

Fig 2: KEGG\_GLYCINE\_SERINE\_AND\_THREONINE\_METABOLISM      
 Blue-Pink O' Gram in the Space of the Analyzed GeneSet

  

Fig 3: KEGG\_GLYCINE\_SERINE\_AND\_THREONINE\_METABOLISM: Random ES distribution      
 Gene set null distribution of ES for **KEGG\_GLYCINE\_SERINE\_AND\_THREONINE\_METABOLISM**
